# P-90. Culture negative vs Culture positive Acute Hematogenous Osteomyelitis in Pediatrics: A single study experience

**DOI:** 10.1093/ofid/ofae631.297

**Published:** 2025-01-29

**Authors:** Salma Sadaf, Kevin J Downes, Jeffrey S Gerber

**Affiliations:** Children's Hospital of Philadelphia, Brooklyn, New York; Children's Hospital of Philadelphia, Brooklyn, New York; Children's Hospital of Philadelphia, Brooklyn, New York

## Abstract

**Background:**

Acute hematogenous osteomyelitis (AHO) is a rare but serious infection in children that can result in acute and chronic sequelae. Despite guidelines, optimal management continues to be a source of debate, particularly in children without positive cultures. We examined treatment approaches and outcomes in AHO patients stratified by culture positivity.

Consort Flow Diagram of the Study on Culture Negative vs. Culture Positive Acute Hematogenous Osteomyelitis in Pediatrics.

**Figure:**
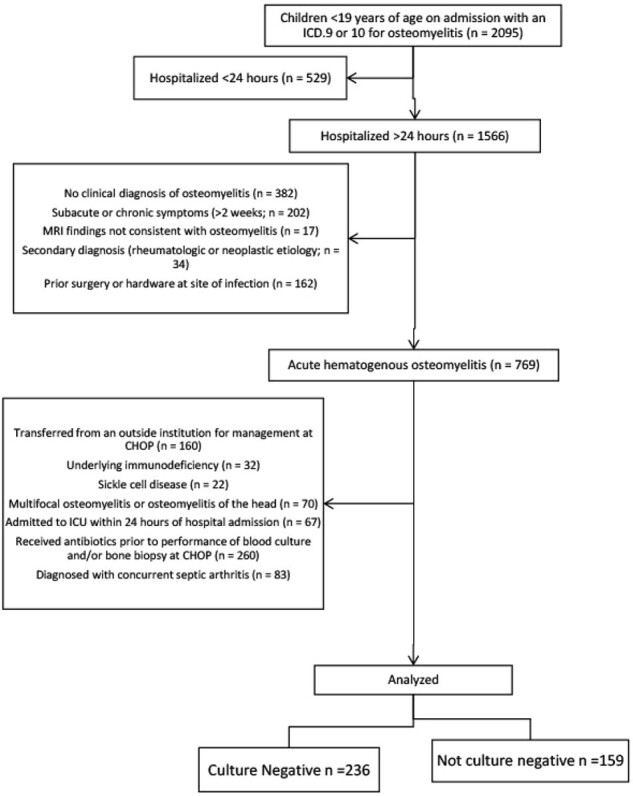

This diagram illustrates the participant flow through each stage of the retrospective study, including enrollment, allocation, follow-up, and analysis. The numbers of participants assessed for eligibility, included in the study, and analyzed for the primary outcome are detailed, alongside reasons for exclusions at each stage.

**Methods:**

This retrospective cohort study included children (< 19y) admitted at CHOP from 2005-2020 with a primary diagnosis of AHO. Cases were identified by APR-DRG and ICD-9 codes followed by chart review to determine inclusion/exclusion criteria (Table 1). Patients were divided into culture-positive (CX+) and culture-negative (CX-) groups. Wilcoxon rank sum tests were used to compare continuous variables and Chi-square test was used to compare categorical variables between groups.

Inclusion/Exclusion criteria for defining AHO cohort

**Figure:**
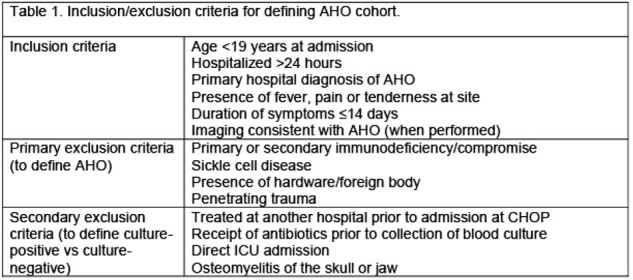

This table details the criteria used to identify pediatric patients for inclusion and exclusion in the Study on Culture Negative vs. Culture Positive Acute Hematogenous Osteomyelitis in Pediatrics. Inclusion criteria focus on patient characteristics and clinical presentation necessary for study entry, including age, hospitalization duration, diagnosis, symptomology, and relevant imaging findings. Exclusion criteria are subdivided into primary factors, which identify general patient disqualifications, and secondary factors, which specifically differentiate between culture-positive and culture-negative cases. These criteria ensure a focused and relevant patient cohort for analysis.

**Results:**

Of 796 children with a primary diagnosis of AHO, 395 [236 (59.7%) culture-negative and 159 (40.2%) culture-positive] met no secondary exclusion criteria. Patient characteristics are shown in Table 2. Blood culture was performed more frequently in CX+ cases (94.3% vs 84.1%, p=0.004), as were bone cultures (54.8% vs. 11.1%, p< 0.001). The proportion of CX- cases decreased over time (2005-8: 66%, 2009-12: 60%, 2013-16: 59%, 2017-20: 57%; p=0.12). The most common organisms identified in CX+ group were MSSA (73.6%) and MRSA (21.4%). CX+ more often received narrow-spectrum beta-lactams empirically (27.5% vs. 10.1%, p< 0.001) and as definitive therapy (60.9% vs 18.0%, p< 0.001). CX+ less often received anti-MRSA antibiotics empirically (65.2% vs. 83.1%, p< 0.001) or as definitive treatment (30.4% vs 78.1%, p< 0.001). CX+ patients had a shorter median hospital stay (4 days) than CX- patients (5 days, p< 0.001). There was no difference in treatment duration, treatment failure or need for repeat surgical intervention.

Features of culture-positive and culture-negative AHO cases

**Figure:**
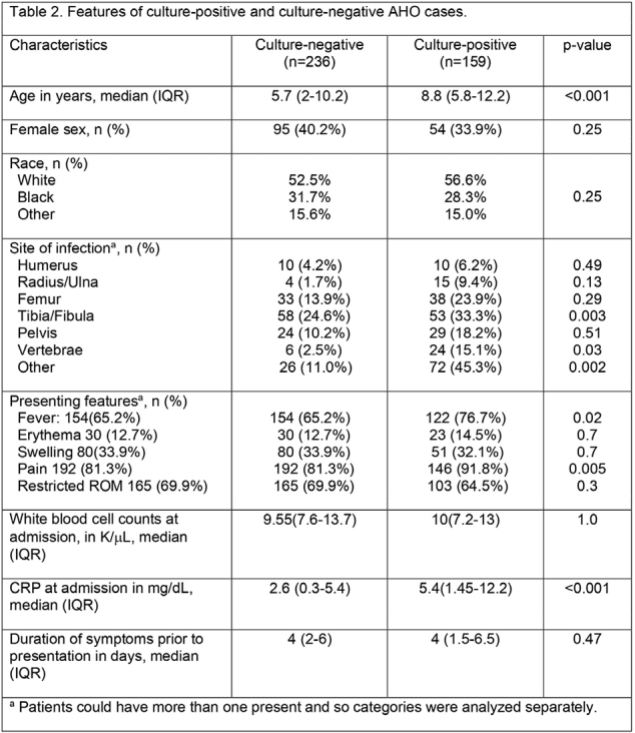

This table summarizes the demographic and clinical characteristics, site of infection, presenting features, and laboratory values at admission for pediatric patients diagnosed with AHO, categorized by culture-positive and culture-negative cases. Data are presented as medians with interquartile ranges (IQR) or as numbers with percentages. Statistical significance between groups is evaluated and shown in the rightmost column as p-values, highlighting differences in infection sites, symptom presentation, and initial laboratory findings between the two groups.

**Conclusion:**

Nearly two-thirds of AHO cases were culture-negative at our hospital. The benefits of obtaining cultures (narrower spectrum therapy, shorter hospital stay) should be weighed since outcomes were similar between these groups.

**Disclosures:**

**Kevin J. Downes, MD**, Paratek, Inc.: Grant/Research Support|Veloxis Pharmaceuticals, Inc.: Grant/Research Support

